# Association of ABO and Rh Blood Group in Susceptibility, Severity, and Mortality of Coronavirus Disease 2019: A Hospital-Based Study From Delhi, India

**DOI:** 10.3389/fcimb.2021.767771

**Published:** 2021-11-02

**Authors:** Rashmi Rana, Vivek Ranjan, Naveen Kumar

**Affiliations:** ^1^ Department of Research, Sir Ganga Ram Hospital, New Delhi, India; ^2^ Department of Blood Transfusion Medicine, Sir Ganga Ram Hospital, New Delhi, India

**Keywords:** ABO and Rh blood groups, susceptibility, severity, mortality, coronavirus disease (COVID-19) outbreak

## Abstract

**Background:**

ABO and Rh blood group systems are associated with many diseases including cancerous, infectious, non-infectious, bacterial and viral diseases. Studies have shown association of blood groups A and O with higher and lower odds for coronavirus disease 2019 positivity, respectively.

**Methods:**

This is a single-center, retrospective study conducted at Sir Ganga Ram Hospital, Delhi. We investigated the association of ABO and Rh blood groups with susceptibility to coronavirus disease 2019 infection, severity of disease, recovery period, and mortality of patients. Patients were enrolled from April 8, 2020 to October 4, 2020. A total of 2,586 real-time PCR (RT-PCR)-confirmed coronavirus disease 2019 (COVID-19) patients were recruited. Data was analyzed using chi-square test, odds ratio, and Mann–Whitney test to determine the association of blood groups.

**Results:**

In the 2,586 COVID-19-infected patients, the frequencies of A, B, O, and AB were 29.93%, 41.80%, 21.19%, and 7.98%, respectively. Of the patients, 98.07% were Rh positive. Blood group A (odds ratio, 1.53; CI, 1.40–1.66; *p* < 0.001) and B (odds ratio, 1.15; CI, 1.06–1.24; *p* < 0.001) is observed to be significantly associated with COVID-19 susceptibility, whereas blood group O (odds ratio, 0.65; CI, 0.59–0.71; *p* < 0.001) and AB (odds ratio, 0.66; CI, 0.59–0.71; *p* < 0.001) have low risk of COVID-19 infection.

**Conclusion:**

A, B, and Rh+ are found to be more susceptible to COVID-19 infection, whereas blood groups O, AB, and Rh− are at a lower risk of COVID-19 infection. No association was found between blood groups and susceptibility to severity of disease and mortality.

## Introduction

Severe acute respiratory syndrome coronavirus 2 (SARS-CoV-2) that emerged from Wuhan, China in December 2019 has posed a great threat to global public health. It causes coronavirus disease 2019 (COVID-19) (Long et al., 2020; Wang et al., 2021). COVID-19 affects people in different ways. It has a wide range of symptoms like fever, dry cough, shortness of breath, muscle pain, fatigue, sore throat, ageusia, and anosmia. However, a large proportion of infected patients remain asymptomatic (Lovato et al., 2020; Rodriguez-Morales et al., 2020). COVID-19 has an incubation period of 1–14 days, but typically, it takes 3–7 days to present symptoms. There are many cases where the COVID-19 infection has taken more than 14 days to present any symptoms (Fan et al., 2020; Huang et al., 2020).

The knowledge of blood group types and their association with COVID-19 may help in disease management and treatment. Out of 34 blood group types that the International Society of Blood Transfusion (ISBT) recognizes, ABO and Rh blood types are the most investigated, studied, and clinically applied. The antigenic structure present on the surface of erythrocytes determines the ABO blood group and also whether there is antigenic structure present or not, determines the Rh system (Sayli, 2020).

Previous studies have found an association between rheumatological diseases, cancers, cardiovascular diseases, infectious and non-infectious diseases, and bacterial and viral diseases, and ABO blood group. Previous studies have shown susceptibility of ABO blood groups to viruses such as Middle East Respiratory Syndrome (MERS), hepatitis B, human immunodeficiency virus, norawalk virus, rotavirus, dengue virus, and SARS coronavirus (Mehta, 2009; Degarege et al., 2012; Chen et al., 2016; Batool et al., 2017; Murugananthan et al., 2018; Wolpin et al., 2010).

Recent studies from China and other parts of the world have reported that there is an association of ABO and Rh blood group with SARS-CoV-2 infection. Blood group type A has high odds of getting infected, while the rate of infection and severity seems less among the blood group O. Rh (D) positive blood group is also associated with increased COVID-19 infection and mortality (Boudin et al., 2020; Dzik et al., 2020; Fan et al., 2020; Goker et al., 2020; Noor et al., 2020; Zietz et al., 2020; Zhao et al., 2021). The underlying mechanism is still unknown and needs to be investigated. Several theories have been proposed to elaborate the mechanism of this association. Genetically encoded blood group antigens might be a predisposing factor for SARS-CoV-2 infection. The human ABO blood group is located on chromosome 9 (9q34.1-34.2) (Amundadottir et al., 2009; Wiggins et al., 2009; Vasan et al., 2016). The ABO blood groups are associated with several bacteria, parasites, and virus infections and also have shown major role as a receptor and coreceptor. ABO blood groups represent a polymorphic trait that has histo-blood group antigens (HBGAs), which are present on the outer surface of red blood cells (RBCs). The expression of HBGAs can decrease or increase the susceptibility of disease (Singh et al., 2016; Liu et al., 2018).

**Figure 1:**
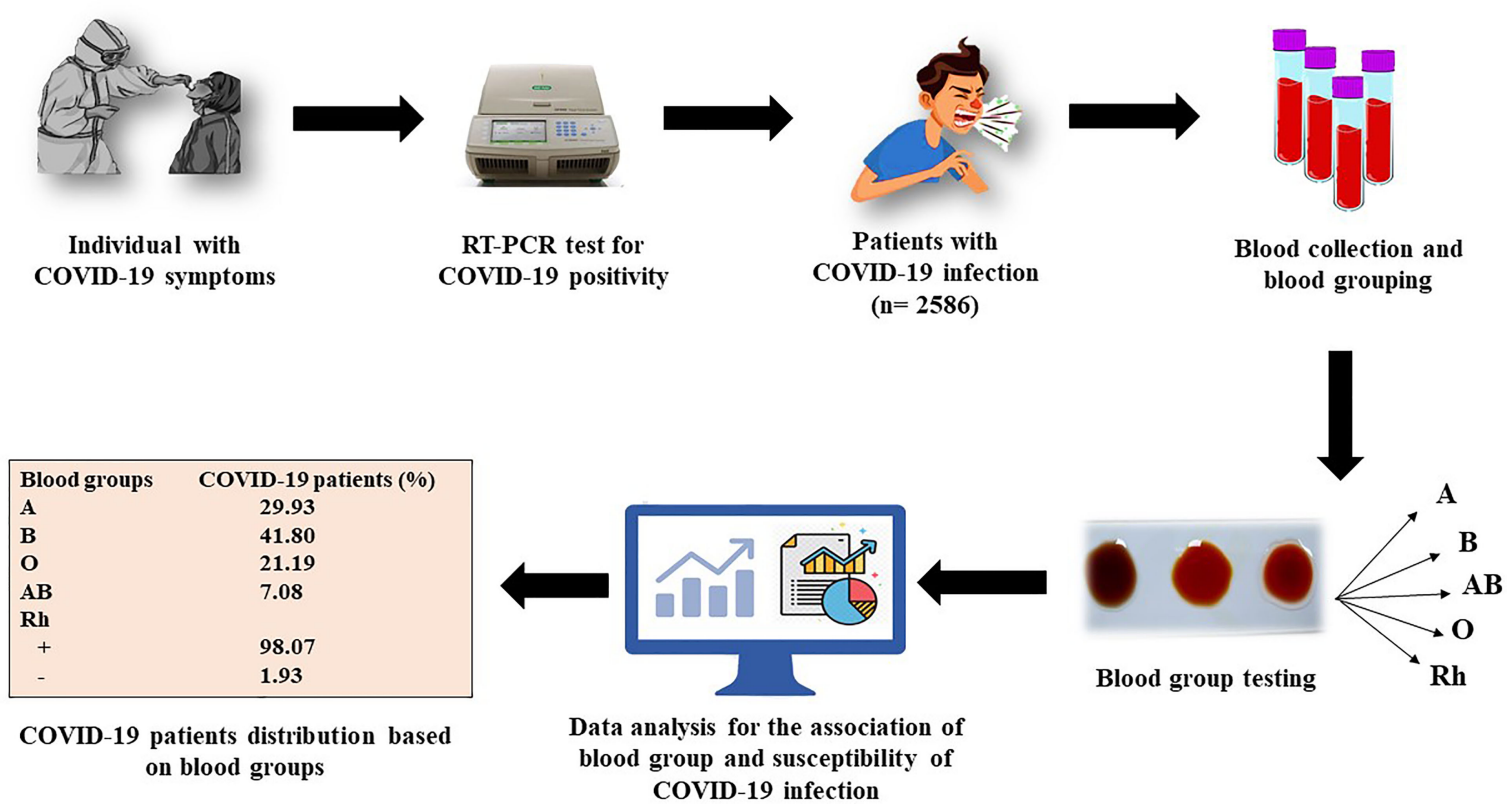
A schematic overview of the study.

Severe acute respiratory syndrome coronavirus 2 is a novel virus, and it is indeterminate whether blood groups have any impact on COVID-19 susceptibility or progression. Therefore, we investigated the association of ABO and Rh blood group with COVID-19 susceptibility, prognosis, recovery time, and mortality in this study. An overview of the study is depicted in **Figure 1**.

## Materials and Methods

### Study Design

This is a single-center, retrospective study that was carried out at Sir Ganga Ram Hospital, Delhi. The Ethical Committee of Sir Ganga Ram Hospital reviewed and approved the study.

We investigated the association of ABO and Rh blood groups with susceptibility to coronavirus disease 2019 infection, severity of disease, length of stay, and mortality of patients. Association was analyzed with ABO blood group and Rh blood group system. This study includes real-time PCR (RT-PCR)-confirmed COVID-19 patients who were admitted to the hospital.

### Case and Control Selection

A total of 2,586 patients with confirmed COVID-19 positivity, tested through real-time PCR for coronavirus disease 2019, were recruited for this study as a case group. All the patients were admitted to Sir Ganga Ram Hospital, Delhi, from April 8, 2020 to October 4, 2020, and follow-up was taken up till their last date of admission as either discharged or deceased.

The control group data were extracted from a review study on blood group distribution in India (Patidar and Dhiman, 2021). The blood group distribution data of Delhi was selected, which was extracted from five independent studies, totalling to 79,325 people (Agarwal et al., 2013; Ahuja et al., 2015; Arora et al., 2015; Garg et al., 2015; Kaur et al., 2016).

### Data Analysis

Statistical Package for Social Sciences (SPSS) Version 18 and MedCalc (statistical software) were used for the analysis. Chi-square test was used to analyze the distribution of ABO and Rh blood groups. Odds ratio (OR) test was applied to study the odds of ABO and Rh blood groups. Odds ratios were reported with 95% confidence intervals (CIs). Mann–Whitney test was used to explore the relationship between recovery period (length of stay at hospital) and blood group. All the tests were applied in a one-vs.-all manner.

## Results

### Susceptibility of COVID-19 Infection

As shown in **Table 1**, the control group is comprised of 79,325 individuals having a high frequency of blood group B, followed by O, A, and AB reported. A total of 2,586 COVID-19-infected patients showed different order of frequency, where blood group B, followed by A, O, and AB were reported as highest to lowest. ABO blood group distribution was statistically different between the two groups (*p* < 0.001). The Rh blood group of the control population was Rh (D) + (n = 73,479, 92.63%) and Rh (D)− (n = 5,846, 7.37%) vs. Rh (D) + (n = 2,536, 98.07%) and Rh (D)− (n = 50, 1.93%) in the COVID-19-infected individuals. Chi-square analysis showed that blood groups A and B were associated with high risk of infections, while blood groups O and AB were associated with a decreased risk of infection. Similarly, Rh+ was found to be associated with increased risk of COVID-19 infection.

**Table 1:** Distribution of ABO and Rh blood groups among controls group and COVID-19-infected patients.

Blood group	COVID-19 patients	Control group	*p*-value	OR (95% CI)
	n = 2,586	n = 79,325		
A	774 (29.93)	17,340 (21.86)	<0.001	1.53 (1.40–1.66)
B	1,081 (41.80)	30,532 (38.49)	0.001	1.15 (1.06–1.24)
O	548 (21.19)	23,298 (29.37)	<0.001	0.65 (0.59–0.71)
AB	183 (7.08)	8,155 (10.28)	<0.001	0.66 (0.57–0.77)
Rh			<0.001	4.04 (3.05–5.35)
+	2,536 (98.07)	73,479 (92.63)		
−	50 (1.93)	5,846 (7.37)		

### Association Analysis by Sex

The COVID-19-infected patients were divided into two groups by sex as shown in **Supplementary Table S1**. Out of 2,586, the male group comprised 1,800 patients. ABO blood group distribution among the two groups showed no difference except for the blood group B. Male patients of blood group B are more prone to COVID-19 than the female patients with blood group B. The association is compared in one-vs.-all blood group manner. Similarly, in comparing Rh blood group, no significant difference was found among the two groups. Chi-square analysis showed that the sex of the patients and ABO and Rh blood groups were not associated with susceptibility of COVID infection

### Association Analysis by Age

Chi-square test was used to compare the blood groups and age groups of the COVID-19 patients. COVID-19-infected patients were divided into two groups as ≤60 and >60 years as shown in **Supplementary Table S2**. Blood group AB was observed to be more susceptible to infection in patients with age group ≤60 years. No other association was observed among other ABO or Rh blood groups.

### Severity of COVID-19 Infection

The severity of COVID-19 is measured based on the admission to intensive care unit (ICU). The association of ABO and Rh blood groups with susceptibility to severity of COVID-19 was analyzed by blood group distributions of COVID-19-infected individuals that required ICU admission (COVID+ ICU+) and those who did not require ICU admission (COVID+ ICU−). The distribution of the two groups is shown in **Table 2**. Chi-square analysis of the blood groups applied on one-vs.-all showed no significant difference. There was no association observed between ABO and Rh blood groups with susceptibility to a severe infection.

**Table 2:** Distribution of ABO and Rh blood groups between COVID+ ICU+ and COVID+ ICU−.

Blood group	COVID+ ICU+	COVID+ICU−	*p*-value	OR (95% CI)
	n = 779	n = 1,807		
A	228 (29.27)	546 (30.22)	0.629	0.96 (0.80–1.15)
B	329 (42.23)	752 (41.62)	0.770	1.03 (0.87–1.22)
O	174 (22.34)	374 (20.70)	0.349	1.10 (0.90–1.35)
AB	48 (6.16)	135 (7.46)	0.234	0.81 (0.58–1.43)
Rh			0.546	0.83 (0.46–1.51)
+	762 (97.82)	1,774 (98.17)		
−	17 (2.18)	33 (1.83)		

### Analysis of Recovery Duration

As shown in **Table 3**, the median (IQR) hospital length of stay (LOS), i.e., the recovery duration, was found to be significantly increased among the patients with blood group O, whereas in patients with blood group A, the median (IQR) LOS was observed to be significantly low when analyzed in manner one-vs.-all using Mann–Whitney test. There was no difference observed in case of patients with blood groups B and AB. Comparing the Rh blood groups, the median (IQR) LOS of patients with Rh (D)+ was significantly lower than that of patients with Rh (D)−.

**Table 3:** Comparison of ABO and Rh blood groups distribution and recovery period (LOS) of patients with COVID-19 infection.

Blood group	Median LOS	IQR	*p*-value
A	9	6–12	<0.001
B	9	7–13	0.858
O	10	7–15	<0.001
AB	10	7–13	0.290
Rh			<0.001
+	9	7–13	
−	11.5	8.25–16	

### Analysis of Risk of Mortality

To test the association of ABO and Rh blood groups with deceased COVID-19 patients, blood groups distribution was compared between deceased and recovered COVID-19-infected patients. There was no significant difference and association found with the ABO and Rh blood groups, as shown in **Table 4**.

**Table 4:** Distribution of ABO and Rh blood groups between deceased and recovered COVID-19-infected patients.

Blood group	Deceased	Recovered	*p*-value	OR (95% CI)
	n = 317	n = 2,269		
A	81 (25.55)	693 (30.54)	0.069	0.09 (0.05–0.17)
B	136 (42.90)	945 (41.65)	0.672	1.05 (0.83–1.34)
O	80 (25.24)	468 (20.63)	0.60	1.30 (0.99–1.71)
AB	20 (6.31)	163 (7.18)	0.569	0.87 (0.54–1.41)
Rh			0.211	0.63 (0.30–1.31)
+	308 (97.16)	2,228 (98.19)		
−	9 (2.84)	41 (1.81)		

## Limitations

The major limitation of this study is that it does not consider underlying factors like comorbidities of the patients. Furthermore, the duration of the study was wide, and the treatment guidelines are dynamic and change over time as per the revised national guidelines, which might have affected the progression of disease.

## Discussion

This is a single-center, retrospective study. The patients visiting to the hospital with COVID-19 symptoms were tested for SARS-CoV-2 positivity *via* RT-PCR test. Blood sample was collected from the COVID-19-positive admitted patients for blood group testing and other pathological tests. Then, the medical history and records were extracted from the hospital database as shown in **Figure 1**.

ABO and Rh blood group types are the most widely used blood groups in clinical studies. The ABO blood group system basically contains two antigens, namely, A and B. The antigen coding gene is located on chromosome 9q34.1 and 9q34.2. It consists of three alleles (A, B, and O) and is four phenotypes (A, B, O, and AB) (Amundadottir et al., 2009; Vasan et al., 2016).

HBGAs are complex molecules present on the surface of RBC membranes. The involvement of complex molecules in modifying the progression of disease is through the action of natural antibodies (Neil et al., 2005; Storry and Olsson, 2009; Ewald and Sumner, 2018). The association between ABO blood groups and diseases have been widely explored for several diseases including viral diseases.

The current study was conducted to observe the association of ABO and Rh blood groups on susceptibility of coronavirus disease 2019 infection, disease severity, recovery time (LOS), and mortality. The study also compares the susceptibility of infection with sex, age, and blood group types. We seek to analyze the association of ABO and Rh blood groups for risk of coronavirus disease 2019 infection among the population in Delhi, India.

A total of 2,586 patients were recruited for the study. In coronavirus disease 2019-infected patients, it was observed that blood groups A and B were more disposed to SARS-CoV-2, whereas blood group O and AB had a significantly lower risk of infection. On comparing the distribution of ABO and Rh blood groups of COVID-19-infected patients with the general population of Delhi, there was a significant increase in infected individuals with blood groups A and B. A significant decrease in the number of COVID-19 patients with blood groups O and AB was also observed.

In a study by Li et al. on 265 SARS-CoV-2 patients, it was observed that blood group A was significantly higher in infected individuals than in the healthy control group population, while blood group O in COVID-19-infected patients was significantly less. Fan et al. from a study on 105 infected patients reported that an individual with blood group A is associated with a high risk of COVID-19 infection, whereas other blood groups had no association (Fan et al., 2020). A study by Zhao et al. on 2,173 COVID-19-infected patients reported that blood group A was higher in COVID-19-infected patients, and blood group O was associated with low risk of infection (Zhao et al., 2021). Similar association of blood group A with increased risk and O with low risk was reported by Solmaz and Araç, who also reported almost a significant increase in risk of infection in patients of blood group AB (Solmaz and Araç, 2021).

Our study also reported similar findings with the association of A and O blood groups. Furthermore, our study adds to the previous results and found that blood group B is also associated with increased risk of coronavirus disease 2019, whereas AB is associated with lower risk of coronavirus disease 2019.

Comparing the Rh (D)+ and Rh (D)− COVID-19-infected individuals with the healthy group, Rh (D)+ individuals are significantly at greater risk of infection when compared to Rh (D) negative individuals.

Esref et al. found a significant difference in Rh blood group system, whereas in a study by Solmaz and Araç on 1,667 patients in the Diyarbakir community of Turkey, no significant difference was found (Esref et al., 2020; Solmaz and Araç, 2021). In our study, comparing the Rh (D)-positive and Rh (D)-negative COVID-19-infected individuals with the control group, a strong association was observed in Rh (D)-positive individuals having a significantly more susceptibility to COVID-19 infection than the Rh (D)-negative individuals.

The current study compared ABO and Rh blood groups distribution and found no association of sex and age groups with the susceptibility of COVID-19 infection, the exception being blood group B, where male patients in the blood group are more disposed to the COVID-19 infection as compared to female patients of same blood group.

ICU-admitted COVID-19 patients were compared to non-ICU-admitted patients to study the association of blood groups and susceptibility to severity of disease. In a study reporting 2,334 COVID-19 patients, Almadhi et al. also found no association between blood groups and severity of disease. In the current study, the Rh blood group was also investigated for association and found to have no association with susceptibility to severe infection (Almadhi et al., 2021).

The recovery period (length of stay at hospital) and blood groups were tested for association. The recovery period of COVID-19-infected individuals was found to be significantly less in patients with blood group A than non-A blood group, whereas patients with blood group O are observed to have significantly greater recovery period than non-O blood group. Rh (D) positivity is also associated with significantly decreased number of days for recovery. Mahmud et al. compared the relation of recovery period in blood group A to non-A blood group. It was found that COVID-19 positivity at 14th day of infection is significantly higher in blood group A. To the best of our knowledge, there are not many studies in the aspect of association of recovery period and blood groups (Mahmud et al., 2021).

In a meta-analysis of 10 studies by Liu et al., it was found that 5 out of 10 studies have analyzed the association between ABO and mortality due to COVID-19 (Liu et al., 2020). It reported that blood group A was associated with significantly increased risk of mortality when compared to non-A blood group. Muniz-Diaz et al. found that blood group A has significantly high risk of mortality as compared to non-A blood groups, whereas blood group O is associated with significantly low risk of mortality (Muniz-Diaz et al., 2021). Our study contrasts with these studies, as we found that ABO and Rh blood groups are not associated with mortality in COVID-19 patients. Similar to the current results, Solmaz and Araç also found that blood groups have no association with mortality of the patients (Solmaz and Araç, 2021).

## Conclusion

Our study has found that blood groups A, B, and Rh+ are more disposed to COVID-19 infection, whereas blood groups O, AB, and Rh− are significantly of lower risk of COVID-19 infection. ABO and Rh blood groups show no impact on the progression of disease and are not associated with susceptibility to severity of disease or mortality. We also found that blood groups A and Rh+ types are associated with a decrease in recovery period, whereas blood groups O and Rh− are associated with increase in recovery period.

However, the ABO and/or Rh blood groups may not be responsible for this association, as these may indicate an unexplored underlying factor like comorbidity. Therefore, larger, multicenter, and prospective studies are needed to ascertain the relationship of between blood groups and SARS-CoV-2.

## Data Availability Statement

The original contributions presented in the study are included in the article/**Supplementary Material**. Further inquiries can be directed to the corresponding author.

## Ethics Statement

The studies involving human participants were reviewed and approved by Institute ethics committee, Sir Ganga Ram Hospital, Delhi. The ethics committee waived the requirement of written informed consent for participation.

## Author Contributions

RR and NK wrote the manuscript and performed data analysis. VR and RR had full access to data in the study and take responsibility of the data integrity. RR and NK take responsibility of the accuracy of data analysis. RR edited the manuscript, and VR is responsible for data collection. All authors contributed to the article and approved the submitted version.

## Conflict of Interest

The authors declare that the research was conducted in the absence of any commercial or financial relationships that could be construed as a potential conflict of interest.

## Publisher’s Note

All claims expressed in this article are solely those of the authors and do not necessarily represent those of their affiliated organizations, or those of the publisher, the editors and the reviewers. Any product that may be evaluated in this article, or claim that may be made by its manufacturer, is not guaranteed or endorsed by the publisher.
