# Supplementary material for: Association of ABO and Rh Blood Group in Susceptibility, Severity, and Mortality of Coronavirus Disease 2019: A Hospital-Based Study From Delhi, India
Source: Front Cell Infect Microbiol. 2021 Nov 2;11:767771. doi: 10.3389/fcimb.2021.767771 (PMC8593001; doi:10.3389/fcimb.2021.767771)
Supplement: Supplementary file 1 [file Table_1.docx]

**Supplementary Table 1: Distribution of ABO and Rh blood groups among males and females**

| **Blood Group** | **Males**  **n= 1800** | **Females**  **n= 786** | **p** |
| --- | --- | --- | --- |
| **A** | 521 (28.94) | 253 (32.19) | 0.098 |
| **B** | 777 (43.17) | 304 (38.68) | 0.033 |
| **O** | 383 (21.28) | 165 (20.99) | 0.870 |
| **AB** | 119 (6.61) | 64 (8.14) | 0.162 |
| **Rh**  **+**  **-** | 1767 (98.17)  33 (1.83) | 769 (97.84)  17 (2.16) | 0.576 |
