# Supplementary material for: Association of ABO and Rh Blood Group in Susceptibility, Severity, and Mortality of Coronavirus Disease 2019: A Hospital-Based Study From Delhi, India
Source: Front Cell Infect Microbiol. 2021 Nov 2;11:767771. doi: 10.3389/fcimb.2021.767771 (PMC8593001; doi:10.3389/fcimb.2021.767771)
Supplement: Supplementary file 2 [file Table_2.docx]

**Supplementary Table 2: Distribution of blood groups among COVID-19 individuals of age ≤60 years and >60 years**

| **Blood Group** | **≤60 years**  **n= 1755** | **>60 years**  **n= 831** | **p** |
| --- | --- | --- | --- |
| **A** | 530 (30.20) | 244 (29.36) | 0.0664 |
| **B** | 718 (40.91) | 363 (43.68) | 0.182 |
| **O** | 368 (20.97) | 180 (21.67) | 0.688 |
| **AB** | 139 (7.92) | 44 (5.29) | 0.015 |
| **Rh**  **+**  **-** | 1716 (97.78)  39 (2.22) | 820 (98.68)  11 (1.32) | 0.121 |
